# Plant virus transmission during seed development and implications to plant defense system

**DOI:** 10.3389/fpls.2024.1385456

**Published:** 2024-05-08

**Authors:** Cesar Escalante, Alvaro Sanz-Saez, Alana Jacobson, Katarzyna Otulak-Kozieł, Edmund Kozieł, Kipling S. Balkcom, Chaoyang Zhao, Kassie Conner

**Affiliations:** ^1^ Department of Entomology and Plant Pathology, Auburn University, Auburn, AL, United States; ^2^ Department of Crop Soil and Environmental Sciences, Auburn University, Auburn, AL, United States; ^3^ Institute of Biology, Department of Botany, Warsaw University of Life Sciences, Warsaw, Poland; ^4^ The United States Department of Agriculture - Agricultural Research Service (USDA-ARS) National Soil Dynamics Lab, Auburn, AL, United States; ^5^ Alabama Cooperative Extension, Auburn University, Auburn, AL, United States

**Keywords:** plant defense mechanisms, plant viruses, seed, small interfering RNAs, vertical transmission, viral suppressors of RNAi

## Abstract

Most plants produce large amounts of seeds to disperse their progeny in the environment. Plant viruses have evolved to avoid plant resistance mechanisms and use seeds for their dispersal. The presence of plant pathogenic viruses in seeds and suppression of plant host defenses is a major worldwide concern for producers and seed companies because undetected viruses in the seed can represent a significant threat to yield in many economically important crops. The vertical transmission of plant viruses occurs directly through the embryo or indirectly by getting in pollen grains or ovules. Infection of plant viruses during the early development of the seed embryo can result in morphological or genetic changes that cause poor seed quality and, more importantly, low yields due to the partial or ubiquitous presence of the virus at the earliest stages of seedling development. Understanding transmission of plant viruses and the ability to avoid plant defense mechanisms during seed embryo development will help identify primary inoculum sources, reduce virus spread, decrease severity of negative effects on plant health and productivity, and facilitate the future of plant disease management during seed development in many crops. In this article, we provide an overview of the current knowledge and understanding of plant virus transmission during seed embryo development, including the context of host-virus interaction.

## 1 Introduction

**Figure 1:**
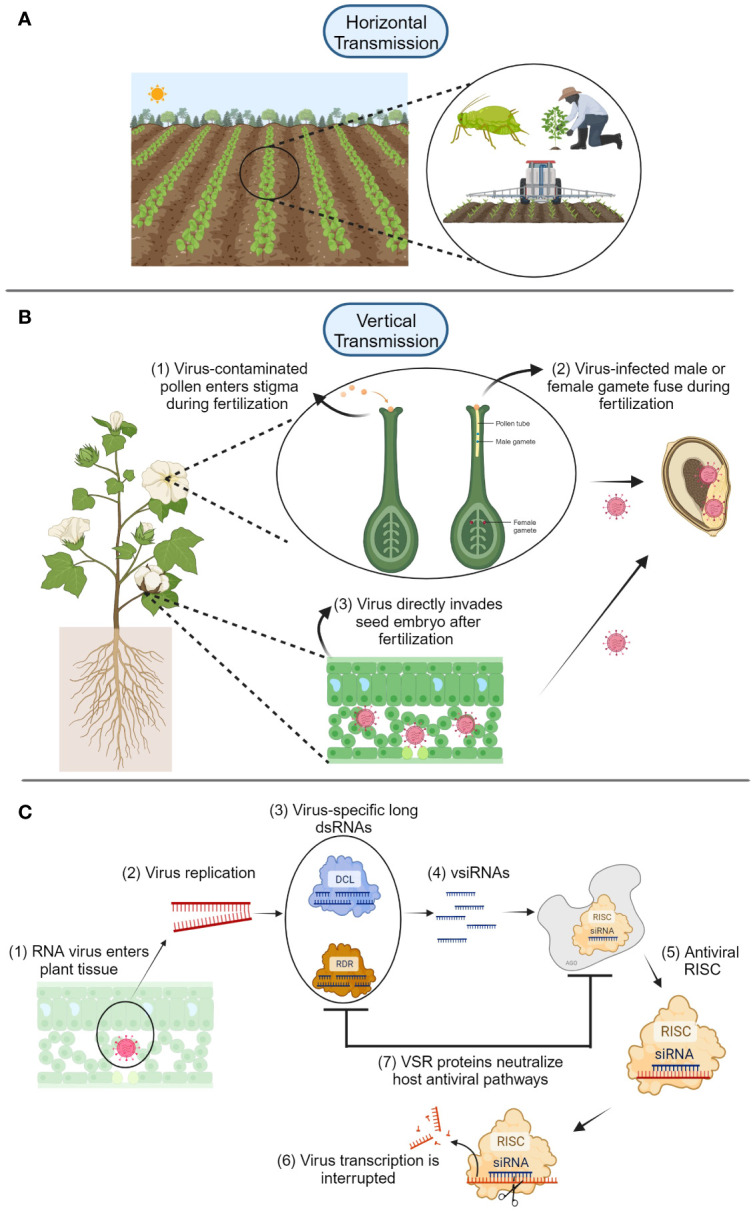
**(A)** Horizontal transmission of plant viruses is mediated by insects, humans, or contaminated agricultural tools. **(B)** Vertical transmission of plant viruses is accomplished through two main routes: embryo invasion via infected pollen or gametes (1-2), and direct infection of the seed embryo via virus movement (3) using virus-encoded proteins such as movement proteins. **(C)** After plant RNA viruses enter the host (1), virus replication begins (2), and Dicer-like (DCL) and RNA-dependent RNA-polymerase (RDR) proteins form complexes with virus-specific long dsRNAs (3) that produce viral small interfering RNAs (siRNA) (4). These complexes are carried by Argonaute (AGO) proteins to form RNA-induced silencing complexes (RISC) that interfere with virus gene transcription and replication (5-6). Viruses can fight back these plant defense mechanisms by using virus suppressors of RNAi (VSR), which can neutralize plant host antiviral pathways (7). Diagrams were created with BioRender.com and are not drawn to scale.

Viruses are obligate parasites/pathogens and depend on the host’s cellular machinery for their replication (Hull, 2014). For this reason, they have different replication strategies that allow latency in a suitable host for infection, as well as replication and movement of the virus particles within the host. Viruses can be naturally transmitted from plant to plant in two ways. The first is horizontal transmission (**Figure 1A**), which can involve assistance from insects, humans, or mechanical spread through agricultural tools or equipment (Maule and Wang, 1996; Hull, 2014). The second is vertical transmission (from plant parent to its offspring, see **Figure 1B**), which consists of virus transmission via seed, pollen, or ovule (Matsushita et al., 2018; Cobos et al., 2019; Pagán, 2022). Seed transmission of plant viruses represents a huge problem for producers and seed companies (Hull, 2014). One main problem is that the presence of infected or infested seeds might go unnoticed, resulting in outbreaks of viral diseases representing a risk for food security. In addition, infection of plant viruses in the early stages of embryo development might cause negative morphological or even genetic effects that result in yield and economic losses.

Depending upon the replication strategy of plant viruses, and the age and resistance level of the host, plant viruses can infect seeds at different stages of development, resulting in progeny infection rates ranging from 0.10 to 92% (**Table 1**). High infection rates of plant viruses have caused many farmers to cease growing various crops due to significant yield losses (Hobbs et al., 2000). The assessment of yield loss is sometimes difficult due to variability in crop yield and damage (Bos, 1982); however, yield loss and damage from plant viruses have been determined using experimental methods that document reductions of physiological parameters such as plant growth and vigor; or commercial and management parameters such as product quality and costs related to disease management (Bos, 1982). Seed transmission plays an important role in survival of viruses from season to season (Johansen et al., 1994). Therefore, transmission of viruses through seed, even at a very low rate, may be important for virus spread, overwintering, and long-range dissemination (Ali and Kobayashi, 2010). In the case of viruses that are vector-transmitted, seed transmission provides an initial source of virus inoculum that may have a considerable impact on crop yield (Ali and Kobayashi, 2010). Several plant viruses present high rates of transmission through seed (Hull, 2014). However, for many plant viruses, evidence of seed transmission is lacking or uncertain. There is limited information regarding host genes that might be involved in preventing virus transmission through seed, or on the contrary, genes that can facilitate the transmission of plant viruses to the seed. Attempts to identify sources of resistance to seed transmission have been studied in crops such as soybean, barley, bean, and the model organism *Arabidopsis thaliana* (Carroll, 1979; Bowers and Goodman, 1982; Wang et al., 2010; Carrillo-Perdomo et al., 2019; Montes et al., 2021). Understanding transmission mechanisms of plant viruses, especially through seed as a primary inoculum, helps reduce viral spread and the severity of negative effects from plant viruses in many crops. The objective of this mini-review was to provide a brief description of molecular mechanisms of plant virus transmission through seed, particularly during reproductive organ development and within-host virus movement.

**Table 1:** Percent seed transmission of selected plant viruses in economically important crops.

Crop	Virus name	Percent of transmission	Source
Black gram (*Vigna mungo*)	Mung vein yellow mosaic virus	32.0	(Kothandaraman et al., 2016)
Cacao (*Theobroma cacao*)	Cocoa swollen shoot virus	40.0 - 53.0	(Quainoo et al., 2008)
Corn (*Zea mays*)	Sugar cane mosaic virus	4.81	(Li et al., 2007)
Corn (*Zea mays*)	Maize chlorotic mottle virus	0.0 - 0.57	(Kimani et al., 2021)
Corn (*Zea mays*)	High plains virus	0.10 - 0.11	(Forster et al., 2001)
Papaya (*Carica papaya*)	Papaya meleira virus	81.20	(Tapia-Tussell et al., 2015)
Pepper (*Capsicum annum*)	Cucumber mosaic virus	10.0 - 14.0	(Ali and Kobayashi, 2010)
Raspberry (*Rubus idaeus*)	Raspberry bushy dwarf virus	13.0	(Isogai et al., 2015)
Soybean (*Glycine max*)	Soybean mosaic virus	25.7 - 91.7	(Pacumbaba, 1995)
Soybean (*Glycine max*)	Soybean vein necrosis virus	6.0	(Groves et al., 2016)
*Sweet potato* (*Ipomoea batatas*)	Sweet potato leaf curl virus	70.0	(Kim et al., 2015)
Tomato (*Solanum lycopersicum*)	Tomato brown rugose fruit virus	2.80	(Davino et al., 2020)
Wheat (*Triticum* spp.)	Wheat streak mosaic virus	0.22	(Lanoiselet et al., 2008)
Zucchini (*Cucurbita pepo*)	Zucchini yellow mosaic virus	21.9	(Simmons et al., 2013)

## 2 Seed infection during development

Approximately one-third of plant viruses are seed-transmitted (Maule and Wang, 1996; Hull, 2014; Otulak et al., 2016), causing significant economic consequences in many crops. The presence of plant viruses in seeds might severely affect plant development at very early stages, rendering cell ultrastructure malformation during seedling development and impairing functions of cellular structures such as chloroplast that play an important role in photosynthesis (Harsányi et al., 2002). This impacts the seed industry, which is required to produce virus-free seeds for market release.

The suspensor, which is a long line of cells supporting the developing embryo, is considered one route for direct viral invasion of the embryo; this occurs because the suspensor is one of the first tissues to be infected (Wang and Maule, 1994; Bewley et al., 2013). During the programmed degeneration of the suspensor during embryo development, there may be a transient window for invasion of the embryo (Wang and Maule, 1994), and that is the reason for having a temporal and spatial pattern of accumulation and transmission of plant viruses in the embryo. For instance, the presence of sugar cane mosaic virus (SCMV) in maize depends upon the developmental period of the embryo (Li et al., 2007). Li et al. (2007) summarized the time course of SCMV detection in maize kernels; they noticed that the infection rate might be related to the age of the host. This indicates that late infection of some viruses may not cause transmission through seed due to the absence of the suspensor at this stage of embryonic development.

Although many viruses can be transmitted through seeds, they do not necessarily infect the seed embryo. For example, the well-known group of tobamoviruses, now arranged in the family *Virgaviridae* (Hull, 2014), causes contamination of the seed coat, which results in a subsequent infection in the germinating seedling (Isogai et al., 2015). However, these viruses can be easily eliminated by different seed treatment methods (i.e., thermotherapy and chemotherapy) as the virus particles infest only the surface of the seed. Among the seed treatment methods, sodium hypochlorite is very common due to its effectiveness and availability. In a recent study, using sodium hypochlorite helped to successfully eliminate apple chlorotic leaf spot virus, apple stem grooving virus, and apple stem pitting virus from the seed coat of maternally-infested apple seeds (Wunsch et al., 2024). Virus particles have also been found in various types of reproductive organs having a negative effect on the seed and pollen development of both tobacco and pepper (Otulak et al., 2016). Performing ultrastructural analyses, Otulak et al. (2017) demonstrated that virions and inclusion bodies of the necrotic strain of potato virus Y were detected in *Capsicum annuum* (cv Yolo Wonder) reproductive organs including anthers, ovaries, pollen grains, and pollen tubes (**Figure 1B**). Once the virus reaches the embryo, it has different distribution patterns. In peas, the viral RNA tends to be more distributed in the carpel and integuments in ovary tissues (Wang and Maule, 1994). In immature pea seeds infected with pea seed-borne mosaic virus (PSbMV), the distribution of the virus is located in the testa tissue. Interestingly, the amount of virus particles seems to decrease as seeds mature and distribution of PSbMV is reduced and limited to patches in the testa of older seeds (Wang and Maule, 1994). This pattern may be specific to the type of virus, due to different viral replication strategies, or it might also depend on the host species. In-host virus movement modeling studies suggested lesser virulent viruses might have higher chances of being vertically transmitted (Cobos et al., 2019). This finding suggests a more passive virus-host interaction that seldom activates plant defense mechanisms. Similarly, the effective size of population analysis of PSbMV has demonstrated that seed-transmitted viruses undergo high-level genetic drift during seed transmission compared to horizontal leaf transmission of viruses (Fabre et al., 2014).

Transmission of plant viruses involves different physical and biological properties of both the virus and the host. Maule and Wang (1996) described biological characteristics of seed transmission of plant viruses. They arranged these characteristics in seven sections as follows: *i*) different cultivars of the same host species may vary in their seed transmission of a single virus isolate, perhaps due to different genetic compositions rendering in different levels of antiviral defense mechanisms; *ii*) transmission of different isolates of the same virus varies in a single host cultivar; *iii*) efficiency of transmission is environmentally influenced; *iv*) seed transmission seldom reaches 100%; *v*) age of the mother plant might influence seed transmission; *vi*) except for tobamoviruses, in most cases it is necessary for the embryo to be infected; and *vii*) seed transmission mostly occurs through infection of the gametes before fertilization, although there might be infection of the embryo after fertilization.

## 3 Molecular mechanisms of virus seed transmission and plant defense

A seed is structurally composed of several parts: the seed coat, the embryo (including the embryonic axis and one or two cotyledons) (Bewley et al., 2013), and often accompanied by a nutritive tissue, the endosperm, which supports seedling development. The embryo is an important linkage in the transmission of plant viruses because it is structurally connected to other plant tissues via the suspensor, especially during the early stages of seed development (Bewley et al., 2013). These parts can be easy to distinguish in dicotyledonous plants such as soybean and cotton but can be more difficult to distinguish in monocotyledonous plants such as corn and wheat because some parts of the cotyledon undergo structure modifications to form special structures such as the scutellum, coleoptile, radicle, and coleorhiza (Bewley et al., 2013). Viruses depend solely on vectors or mechanical means for transmission from plant to plant (horizontal transmission). On the other hand, the cell-to-cell movement of plant viruses within the plant is via plasmodesmata. This virus movement occurs within the host regardless of whether it is vertically or horizontally transmitted. During this process, viruses reach vascular tissues and move long distances with the aid of movement proteins (Roberts et al., 2003). The latter mode of virus transmission through seed has been suggested to be one of the most efficient means to measure the percentage of seed transmission of plant viruses (Cobos et al., 2019).

Two physical routes for seed embryo invasion of plant viruses have been proposed: *i*) a direct invasion of the host embryo after fertilization and *ii*) an indirect invasion of the zygote during fertilization from previously infected gametes (Johansen et al., 1994; Wang and Maule, 1994; Maule and Wang, 1996; Wang and Maule, 1997; Bradamante et al., 2021) (**Figure 1B**). When these two embryo infection routes were proposed, little information was known about the fate and persistence of viruses in the embryo. Studies investigating host plant resistance to seed entry/transmission have provided information about mechanisms responsible for vertical transmission of viruses. Carroll’s (1979) initial study on the host genetic basis for the interaction between barley stripe mosaic virus and barley suggested that a single recessive gene was involved in the regulation of seed transmission. However, very little was known about other mechanisms involved in transmission of plant viruses through seed and the gene itself. Bowers and Goodman (1982) screened soybean lines to identify resistance to seed transmission of soybean mosaic virus (SMV). Although they reported soybean lines resistant to seed transmission of SMV, molecular bases of resistance were not known.

Recent research has demonstrated that several genes are involved in the control of seed transmission. For instance, chromosomal regions on linkage groups can facilitate seed transmission of SMV in soybean embryos (Domier et al., 2011). The researchers suggested that chromosomal regions associated with seed transmission of SMV contain homologies to *Arabidopsis thaliana* genes like DCL3 and RDR6, which code for proteins Dicer-like and plant RNA-dependent RNA polymerase 6, respectively (**Figure 1C**). These two enzymes can have antiviral effects in plants in the form of transcriptional and posttranscriptional gene silencing by a combination of viral small interfering RNAs (vsiRNA) and protein aggregations called RNA-induced silencing complex (RISC) (Wang et al., 2010; Guo et al., 2019; Bradamante et al., 2021). Perhaps one of the most recent studies showing how a single gene can change the fate of resistance to seed transmission was published by Matsushita et al. (2024). In their study, they tested several cultivars of three Solanaceous crops (tomato, bell pepper, and eggplant) with two resistant genes to tomato brown rugose fruit virus (ToBRFV), *Tm-2^2^* and *L^3^*. They found that bell pepper (cv Miogi) containing the *L^3^* gene produced seeds without detectable levels of ToBRFV; however, these results were not consistent among species and cultivars, confirming that genes conferring resistance to seed transmission have different outcomes depending upon the overall host’s genetics.

Different mechanisms and rates of virus transmission through seeds have been observed depending upon specific virus host interactions and the phenological stage of the host (**Table 1**). Transmission of some viruses (i.e., potyviruses) depends on the stage of seed development, for example, pea seed-borne mosaic virus (PSbMV) is transmitted during the early stage of seed development in pea seeds (Roberts et al., 2003). It was recently shown that PSbMV protein HC-Pro, acts as viral suppressors of RNAi (VSR) and mediates vertical transmission by neutralizing the host antiviral RNAi pathway through Argonaute (AGO) protein depletion during early seed development (Johansen et al., 1996; Incarbone and Dunoyer, 2013; Csorba et al., 2015; Bradamante et al., 2021) (**Figure 1C**). Other VSR proteins such as 12K of pea early browning virus and γb of barley stripe mosaic virus can play important roles in viral infection (Edwards, 1995; Wang et al., 1997). Using GWAS analysis, several genes including *HSP20*-like, *ZAT8*, and *LURP1*, *GMD1*, *PLL18*, *P4H11*, *RTFL13*, *ORTHL*, *CIPK2*, and *MAC5C*, related to biotic and abiotic stress response, were identified in *A. thaliana* after infection of cucumber mosaic virus (Montes et al., 2021). These genes are involved in cell wall metabolism and response to abiotic and biotic stresses from pathogens and their overexpression made the authors hypothesize that they could play an important role in regulating virus transmission to the progeny, although further functional analysis needs to be performed to confirm these results. These preliminary data indicate that biotic or abiotic stress-related genes play an important role in changing and perhaps strengthening seed structures to counteract virus movement to seed structures (Montes et al., 2021). The identification of more genes involved in virus transmission to the embryo, as well as the elucidation of the respective metabolic pathways of gene products, will help develop cultivars with inserted genes and prevent or attenuate transmission of plant viruses to the seed embryo.

Research has been conducted to understand transmission of plant viruses through seeds and thus develop a concise way to control this form of virus transmission. Many genes are potentially involved in preventing transmission of plant viruses through seeds (Wang and Maule, 1994; Montes et al., 2021). Wang and Maule (1994) suggested that seed-transmitted virus in peas and other legumes, such as pea seed-borne mosaic virus, invades pea embryos early in development. They suggested that the infection process is controlled by maternal genes and found a cultivar (cv Progreta) that shows no seed transmission (Wang and Maule, 1994). In this case, virus transmission may have been prevented through the action of multiple host genes segregating as quantitative trait loci. These genes might control the ability of the virus to spread into and multiply in nonvascular testa tissues, hence, preventing the virus from crossing the link between maternal and progeny tissues (Wang and Maule, 1994).

## 4 Future directions

Understanding the transmission of plant viruses in different stages of embryo development will help to better control plant viruses. Similarly, the identification of more host genes involved in the prevention of invasion of plant viruses in the embryo, as well as the identification of metabolic pathways of gene products, will aide in development of resistant cultivars with genes that prevent virus transmission to the seed embryo. Despite numerous research to characterize viruses transmitted through seed, few studies have been performed to understand physiological and molecular host-virus interactions during seed transmission. This leaves a gap in knowledge on the biology of seed transmission, making it a complex topic. However, advancements in next-generation sequencing technologies that allow studying specific sites or tissues (i.e., spatial transcriptomics and single-cell sequencing) rather than bulk tissues will possibly help to better understand plant defense mechanisms to control seed-transmitted viruses that can be further implemented in breeding programs.

## Author contributions

CE: Conceptualization, Funding acquisition, Methodology, Visualization, Writing – original draft. AS: Writing – review & editing. AJ: Funding acquisition, Writing – review & editing. KO: Writing – review & editing. EK: Writing – review & editing. KB: Funding acquisition, Writing – review & editing. CZ: Writing – review & editing. KC: Funding acquisition, Writing – review & editing.
